# Serum vitamin levels in multiple system atrophy: A case-control study

**DOI:** 10.3389/fnagi.2022.1105019

**Published:** 2023-01-05

**Authors:** Daji Chen, Linlin Wan, Zhao Chen, Xinrong Yuan, Mingjie Liu, Zhichao Tang, You Fu, Sudan Zhu, Xuewei Zhang, Rong Qiu, Beisha Tang, Hong Jiang

**Affiliations:** ^1^Department of Neurology, Xiangya Hospital, Central South University, Changsha, China; ^2^Key Laboratory of Hunan Province in Neurodegenerative Disorders, Central South University, Changsha, China; ^3^Department of Radiology, Xiangya Hospital, Central South University, Changsha, China; ^4^Hunan International Scientific and Technological Cooperation Base of Neurodegenerative and Neurogenetic Diseases, Changsha, China; ^5^National Clinical Research Center for Geriatric Disorders, Xiangya Hospital, Central South University, Changsha, China; ^6^Health Management Center, Xiangya Hospital, Central South University, Changsha, China; ^7^School of Computer Science and Engineering, Central South University, Changsha, China; ^8^School of Basic Medical Science, Central South University, Changsha, China; ^9^National International Collaborative Research Center for Medical Metabolomics, Central South University, Changsha, China

**Keywords:** multiple system atrophy, vitamins, Parkinson’s disease, pathogenesis, biomarker

## Abstract

**Aim:**

There is increasing evidence suggesting that vitamins may play important roles in the pathogenesis of multiple system atrophy (MSA). The purpose of this study was to detect the changes of serum vitamin levels and investigate their correlation with disease severity in MSA patients.

**Methods:**

In this cross-sectional study, 244 MSA patients, 200 Parkinson’s disease (PD) patients and 244 age-gender matched healthy controls were recruited. Serum vitamin levels were measured, including vitamin A, B1, B2, B9 (folate), B12, C, D, and E. Relevant clinical scales were used to assess the disease severity of MSA patients.

**Results:**

Compared with the healthy controls, decreased serum folate levels and increased serum vitamin A and C levels were detected in MSA patients. Similar differences were also observed in the gender-based subgroup analysis. There were no differences detected between MSA and PD patients. In MSA patients, significant correlation was found between vitamin A, folate, or vitamin C and relevant clinical scales or laboratory findings. In addition, ROC analysis showed potential diagnostic value of the combination of vitamin A, folate, and vitamin C in distinguishing MSA patients from healthy controls.

**Conclusion:**

There were significant changes in the blood vitamin spectrums of MSA patients, suggesting that dysregulation of vitamins homeostasis might play an important role in the pathogenesis of MSA.

## 1. Introduction

Multiple system atrophy (MSA) is a rare but fatal neurodegenerative disease characterized by variable combination of progressive autonomic dysfunction, Parkinson’s symptoms, cerebellar ataxia, and pyramidal tract dysfunction, with a mean survival time being 6–10 years from symptom onset (Wenning et al., 2013; Coon et al., 2015; Foubert-Samier et al., 2020). Varied clinical presentations are the results of different pathologies including striatonigral, olivopontocerebellar, and central autonomic degeneration. Clinically, MSA is mainly divided into two subtypes, the parkinsonism subtype (MSA-P) and the cerebellar subtype (MSA-C), according to the predominant motor symptom or the onset sign (Krismer and Wenning, 2017; Poewe et al., 2022). The prominent pathological feature of MSA is oligodendroglia cytoplasmic inclusions (GCIs) mainly composed of α-synuclein (Ahmed et al., 2012; Koga et al., 2021), which is also the essential histologic hallmark for diagnosis of definite MSA (Gilman et al., 2008). MSA imposes an immense burden on patients and families. However, little is known about therapies for MSA due to the insufficient understanding of both the etiological factors and the pathological mechanisms influencing disease progression (Fanciulli and Wenning, 2015). Clinical diagnosis is also quite challenging because of significant overlap of clinical symptoms with other movement disorders, particularly Parkinson’s disease (PD) and other parkinsonian disorders (Stankovic et al., 2019). Therefore, reliable biomarkers are crucial for both diagnosis and treatments of patients.

Vitamins are a group of essential organic compounds playing important roles in the regulation of metabolism, growth and development, as well as maintenance of life (de Vries Jasmijn et al., 2018). There is abundant evidence that kinds of vitamins levels in blood varied across individuals with neurodegenerative disease (Blasko et al., 2021; Schulz et al., 2021; Rahnemayan et al., 2022). Numerous studies have been performed to explore the association between vitamins and the risk of PD as well as the role of vitamin supplementation in the prevention and treatment of PD (Lehmann et al., 2017; Schirinzi et al., 2019; Christine et al., 2020; Zhong et al., 2022). Recent metabolomic studies of cerebrospinal fluid indicated that some kinds of vitamins were associated with age and neurodegenerative diseases (Hwangbo et al., 2022a,b). Since MSA shares neuropathogenesis similarities with PD, the exact roles of vitamins in MSA remain to be investigated. Although several studies have analyzed the alternation of vitamins in MSA, the findings in relative small size were controversial (Chen et al., 2015; Zhang et al., 2015; Guo et al., 2017), making it necessary to validate the association of vitamins with MSA in the larger cohorts.

Our study enrolled the largest MSA cohort so far, and investigated the difference of serum vitamin spectrums among MSA patients, PD patients and healthy controls (HC). Furthermore, we evaluated the correlations between serum levels of different vitamins and disease severity in MSA patients. Our study aimed to explore the dysregulation of vitamins homeostasis in MSA patients, as well as their potentials as biomarkers for MSA.

## 2. Materials and methods

### 2.1. Patients and study design

A hospital-based case control study was performed in the Xiangya Hospital of Central South University. 244 MSA patients and 200 PD patients were enrolled from Department of Neurology of Xiangya Hospital between March 2017 and October 2021. Patients with MSA were divided into two subtypes (MSA-P and MSA-C) according to the consensus criteria for the clinical diagnosis of both probable and possible MSA which was established in 2008 (Gilman et al., 2008). The patients with PD fulfilled the MDS Clinical Diagnostic Criteria for Parkinson’s Disease, respectively (Postuma et al., 2015). Additionally, we recruited 244 gender-age matched healthy control subjects from the Physical Examination Center of the same hospital. Exclusion criteria were as follows: (1) gastrointestinal disorders or gastrointestinal surgery impairing vitamin absorption; (2) pernicious anemia; (3) alcohol abuse or alcohol dependence; (4) other neurodegenerative diseases; and (5) the use of proton pump inhibitors, H2 antagonists, or vitamin supplements. This study was approved by the Ethics Committee of the Xiangya Hospital of Central South University. All subjects agreed and signed informed consent.

### 2.2. Measurement of serum vitamins

Fasting peripheral venous blood was collected from subjects. Then, the serum levels of vitamins (including vitamin A, B1, B2, B9 (folate), B12, C, D, and E) were examined in the Clinical Laboratory of Xiangya hospital by electrochemistry method (LK3000V, Lanbiao, Tianjin, China).

### 2.3. Clinical investigations

A thorough neurological examination was performed on all subjects by two experienced neurologists. Among them, 124 MSA patients were evaluated through clinical scales including the Unified MSA Rating Scale (UMSARS), Hoehn and Yahr Parkinson’s disease staging scheme (H&Y stage), Scales for Outcomes in Parkinson’s Disease-Autonomic (SCOPA-AUT), Mini-Mental State Examination (MMSE) and Frontal Assessment Battery (FAB). UMSARS included four parts (I. Activities of Daily Living; II. Motor Examination Scale; III. Orthostatic hypotension; and IV. Disability Scale) and total UMSARS score was the sum of parts I and II. Hoehn and Yahr Parkinson’s disease staging scheme (H&Y stage) was used to assess overall disease severity (Wenning et al., 2004). The Scales for Outcomes in PD-Autonomic (SCOPA-AUT) questionnaire was used to evaluate the autonomic dysfunction, which covered six different autonomic domains: gastrointestinal, urinary, cardiovascular, thermoregulatory, pupillomotor, and sexual domains (Damon-Perrière et al., 2012). Wexner score was used to evaluate the anal incontinence and constipation specifically. The Frontal Assessment Battery (FAB) score and Mini-Mental State Examination (MMSE) score was used to assess global cognition. In addition, urine residue and serum homocysteine (HCY) levels were also recorded.

### 2.4. Statistical analysis

Continuous data were presented as mean ± standard deviation (SD). Normality assumptions were verified by the Shapiro–Wilk test, and homogeneity of variances were checked by the Levene test. All categorical variables, such as gender and subtype, were presented as percentages.

When the data conformed to the normal distribution, Student’s t test and one-way analysis of variance (ANOVA) were used for comparison among groups. For non-parametric data, Mann–Whitney *U* test and Kruskal–Wallis test were conducted for comparison among two or more groups, respectively. Bonferroni’s *post hoc* analysis was performed for multiple testing and protection against a false positive error. Categorical variables were analyzed using a chi-square test. In subgroup analysis, binary logistic regression model was used to access the odds ratio (OR) and adjust for covariates such as age and gender. Correlations between the clinical characteristics and serum vitamin levels were performed using Spearman’s rank correlation. The diagnostic accuracy of different vitamins and their combination for MSA was calculated by receiver operating characteristic (ROC) curves. A value of *p* < 0.05 was considered to be statistically significant. All statistical analyses were performed through SPSS 26.0 (IBM Corp., New York, NY, United States) and GraphPad Prism 9.0(GraphPad Software, Inc.).

## 3. Results

### 3.1. Characteristics of subjects

Table 1 presented the demographic features and serum vitamin levels of the subjects. This cross-sectional study enrolled 244 MSA [145 males (59.4%) and 99 females (40.6%)], 200 PD patients [113 males (56.5%) and 87 females (43.5%)], and 244 healthy subjects [145 males (59.4%) and 99 females (40.6%)]. The median age of MSA, PD, and healthy controls was 56 (51–63), 56 (52–63), and 55 (52–62), respectively (Table 1). No significant differences were detected in terms of age and gender between MSA patients, PD patients, and healthy subjects. The clinical characteristics of 124 MSA patients were summarized in Table 2, in which the subjects were classified into two subtypes, including 36 MSA-P patients and 88 MSA-C patients.

**Table 1:** Characteristics of the three participant cohorts.

	MSA (mean ± SD)	PD (mean ± SD)	Control (mean ± SD)	χ^2^值	*p*-Value	P_1_	P_2_	P_3_
Number	244	200	244					
Gender				0.501	0.779^a^			
Male (%)	145 (59.4)	113 (56.5)	145 (59.4)					
Female (%)	99 (40.6)	87 (43.5)	99 (40.6)					
Age	56.56 ± 7.55	57.26 ± 7.82	56.06 ± 8.13	3.37	0.185^b^			
Vitamin A (μmol/L)	2.55 ± 0.69	2.48 ± 0.57	2.26 ± 0.16	32.6	<0.001^b^	0.738^c^	<0.001^c^	<0.001^c^
Vitamin B1(nmol/L)	126.78 ± 20.15	125.44 ± 21.27	127.66 ± 14.08	0.719	0.698^b^			
Vitamin B2 (μg/L)	8.00 ± 1.34	8.13 ± 1.42	7.98 ± 1.06	1.94	0.380^b^			
Vitamin B9 (μg/L)	9.74 ± 5.71	10.17 ± 6.09	15.06 ± 5.25	123	<0.001^b^	0.502^c^	<0.001^c^	<0.001^c^
Vitamin B12 (ng/L)	507.98 ± 339.80	488.00 ± 388.81	504.41 ± 217.30	16.1	<0.001^b^	0.181^c^	0.083^c^	<0.001^c^
Vitamin C (μmol/L)	32.12 ± 8.78	32.58 ± 9.50	28.58 ± 6.48	27.5	<0.001^b^	0.968^c^	<0.001^c^	<0.001^c^
Vitamin D (nmol/L)	87.39 ± 28.10	86.59 ± 24.18	87.65 ± 15.56	5.51	0.064^b^			
Vitamin E (μg/ml)	7.05 ± 1.22	6.99 ± 1.23	7.04 ± 0.82	2.70	0.259^b^			

SD, standard deviation; MSA, Multiple System Atrophy; PD, Parkinson’s disease; P_1_, comparison between MSA and PD; P_2_, comparison between MSA and HC; P_3_, comparison between PD and HC.

a Chi-square test.

b Kruskal–Wallis test.

c Bonferroni’s *post hoc* analysis.

**Table 2:** Demographic, clinical parameters in MSA, MSA-P, and MSA-C patients.

	MSA-ALL	MSA-P	MSA-C
Number	124	36	88
Gender (*n*)			
Male (%)	75	19	56
Female (%)	49	17	32
Age (years)	55.96 ± 7.29	57.86 ± 7.01	55.18 ± 7.30
Age of onset (years)	53.49 ± 7.15	55.00 ± 6.91	52.87 ± 7.19
Disease during (years)	2.47 ± 1.21	2.86 ± 1.29	2.31 ± 1.14
UMSARS (total)	38.23 ± 15.04	44.44 ± 13.75	35.68 ± 14.88
UMSARS I	19.36 ± 7.44	21.42 ± 6.45	18.52 ± 7.68
UMSARS II	18.86 ± 8.47	23.03 ± 8.37	17.76 ± 7.95
UMSARS IV	2.61 ± 1.05	2.61 ± 0.93	2.61 ± 1.11
H&Y	3.51 ± 0.91	3.25 ± 0.96	3.67 ± 0.86
SCOPA-AUT	19.90 ± 8.75	24.25 ± 7.5	17.41 ± 8.49
Digestive	4.92 ± 3.57	6.26 ± 3.64	4.19 ± 3.35
Urinary	7.87 ± 4.34	9.69 ± 3.25	6.82 ± 4.57
Cardiovascular	2.00 ± 1.16	2.17 ± 1.10	1.92 ± 1.20
Thermoregulatory	1.95 ± 1.44	2.53 ± 1.81	1.56 ± 1.00
Pupillomotor	0.19 ± 0.71	0.33 ± 0.68	0.56 ± 0.91
Sexual	5.52 ± 1.22	5.62 ± 1.08	5.46 ± 1.32
Wexner score	6.00 ± 4.26	6.85 ± 4.01	5.56 ± 4.36
MMSE	25.44 ± 3.66	25.45 ± 3.62	25.44 ± 3.70
FAB	11.28 ± 3.64	10.83 ± 3.99	11.53 ± 3.46
Hcy	13.12 ± 3.82	12.99 ± 3.14	13.19 ± 4.20
Urine residue	93.12 ± 136.91	120.74 ± 179.08	81.47 ± 114.35

MSA-ALL, whole MSA cohort; MSA-P, MSA parkinsonism subtype; MSA-C, cerebellar subtype; UMSARS, Unified Multiple system atrophy Rating Scale; PDSS, PD Sleep Scale; H&Y, the modified Hoehn and Yahr staging scale; SCOPA-AUT, the Scales for Outcomes in PD-Autonomic questionnaire; MMSE, mini-mental state examination; FAB, The Frontal Assessment Battery score.

### 3.2. Comparisons of vitamin levels among three groups

**Figure 1:**
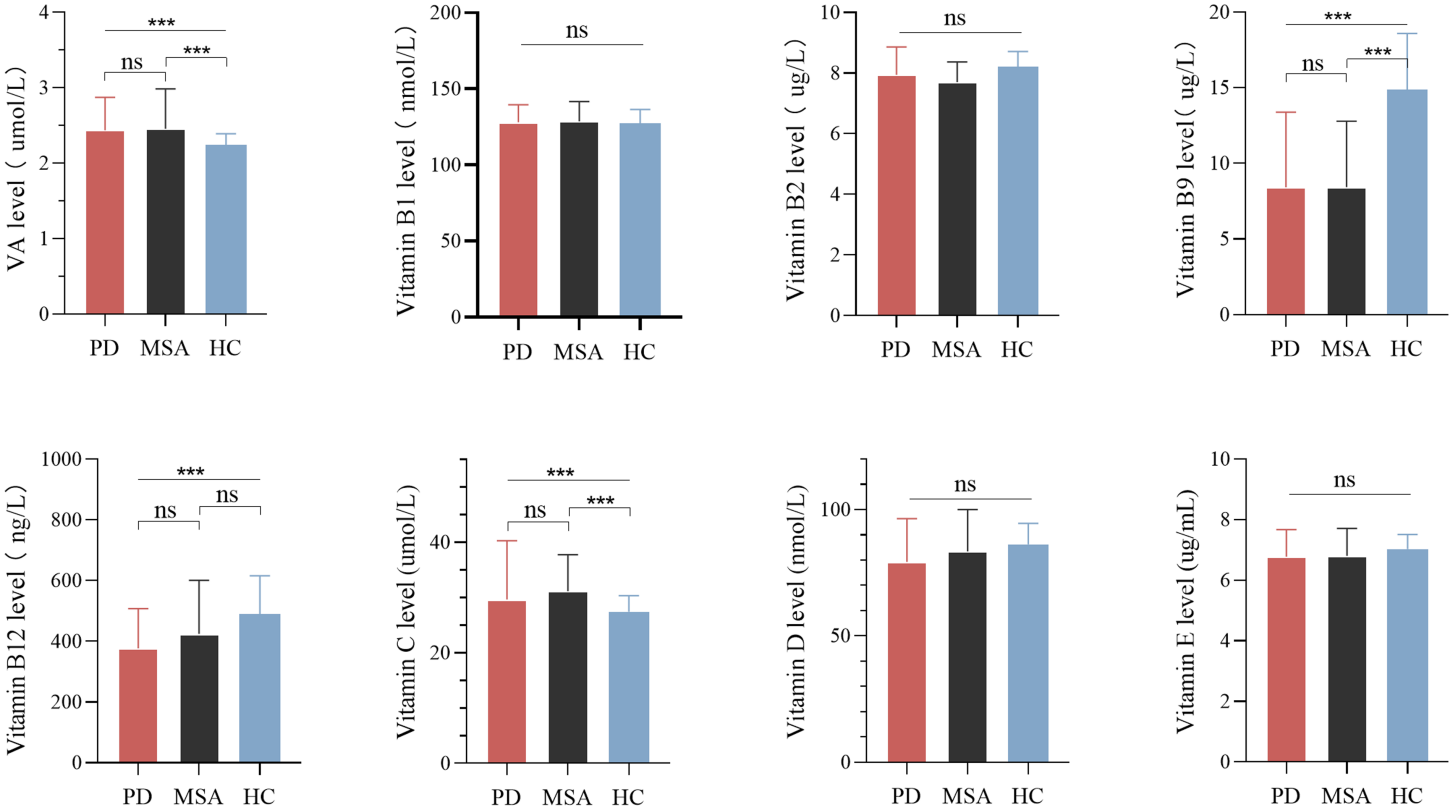
Levels of different vitamins in patients with multiple system atrophy (MSA), Parkinson’s disease (PD), and healthy controls (HC). Multiple comparisons were corrected with Bonferroni correction. ns: non-significant. ***p < 0.001.

There were significant differences in serum vitamin A, B9, B12, and C levels among the MSA, PD and healthy control groups (Table 1; Figure 1). Pairwise comparisons by Bonferroni’s *post hoc* analysis demonstrated that serum vitamin A (*p* < 0.001) and C (*p* < 0.001) in MSA patients were significantly higher than healthy subjects (Table 1), while serum vitamin B9 in MSA was lower than healthy subjects (*p* < 0.001; Table 1). However, for vitamin B12 levels, no significance was identified between MSA and controls, nor that between MSA and PD, while vitamin B12 levels in PD patients were significantly lower than healthy subjects (*p* < 0.001; Table 1; Figure 1). Our results showed no significant differences in the vitamin A, B9, and C levels between MSA and PD patients by Bonferroni’s *post hoc* analysis. In addition, there was no significant difference in serum levels of vitamin B1, B2, B6, D, and E among MSA patients, PD patients, and healthy subjects (Table 1; Figure 1).

**Figure 2:**
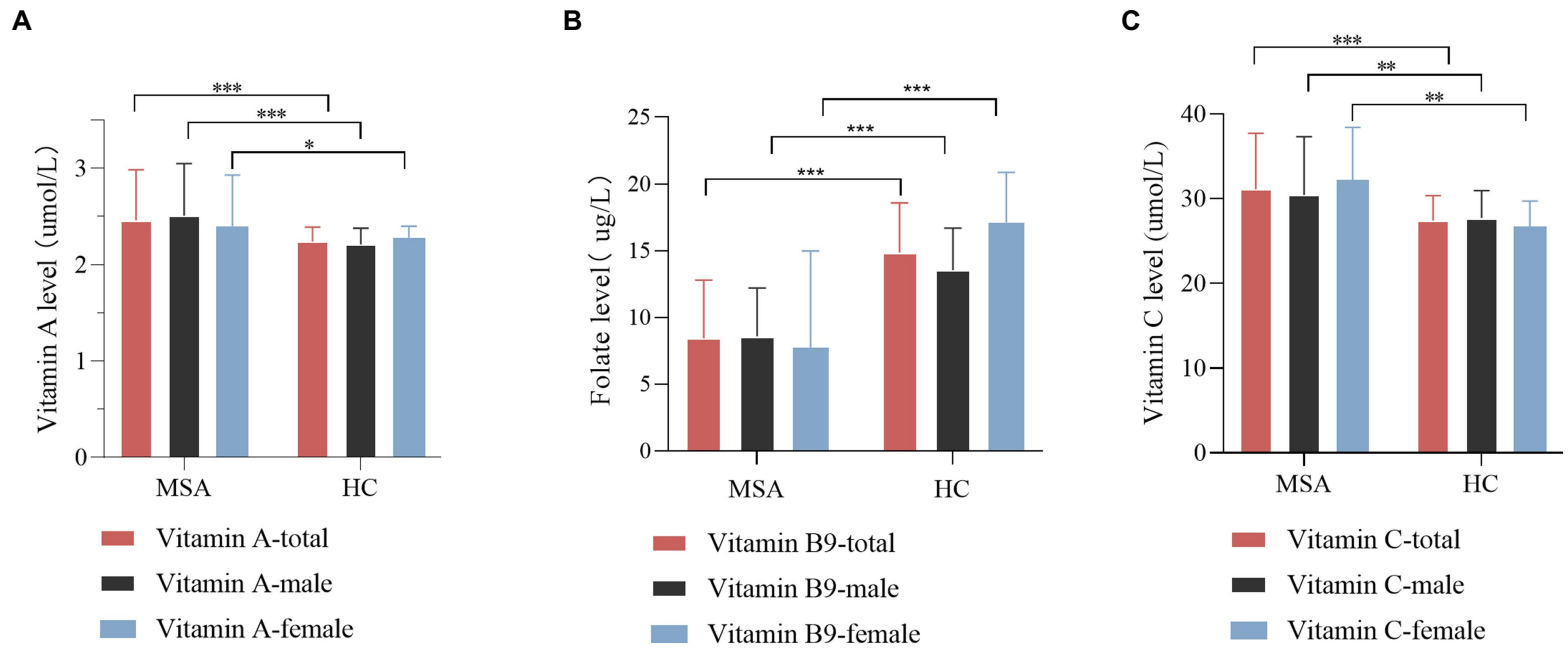
Comparison of vitamin A, B9, and C levels between MSA patients and healthy control (HC), according to gender. **(A)** Comparison of vitamin A level between control and MSA groups. MSA (male) vs. control (male), ***p < 0.001; MSA (female) vs. control (female), *p = 0.024. **(B)** Comparison of vitamin B9 level between control and MSA groups. MSA (male) vs. control (male), ***p < 0.001; MSA (female) vs. control (female), ***p < 0.001. **(C)** Comparison of vitamin C levels between control and MSA groups. MSA (male) vs. control (male), **p = 0.001; MSA (female) vs. control (female), **p = 0.001.

In the subgroup analysis according to gender, similar results retained between MSA patients and healthy subjects (Supplementary Table 1; Figure 2). The vitamin A levels in both male and female subgroup-analysis differed significantly between two groups (*p* = 0.000 for males, *p* = 0.024 for females). Meanwhile, the vitamin C levels exhibited an upward trend in MSA patients than healthy subjects (*p* = 0.001 for males, *p* = 0.010 for females). In addition, regardless of male or female, vitamin B9 levels showed a reduction in MSA patients compared with healthy subjects (*p* < 0.001 for both males and females,). Vitamin B1, B6, B12, D, and E levels did not present any differences in the subgroups (Supplementary Table 1). We also investigated the difference between two subtypes of MSA. Given that age is not equal between the two subgroups, binary logistic regression was utilized to adjust age and gender. The result revealed no significant difference in serum levels of vitamins between two subgroups (Supplementary Table 2).

### 3.3. Correlations between vitamin levels and clinical assessing scales

**Figure 3:**
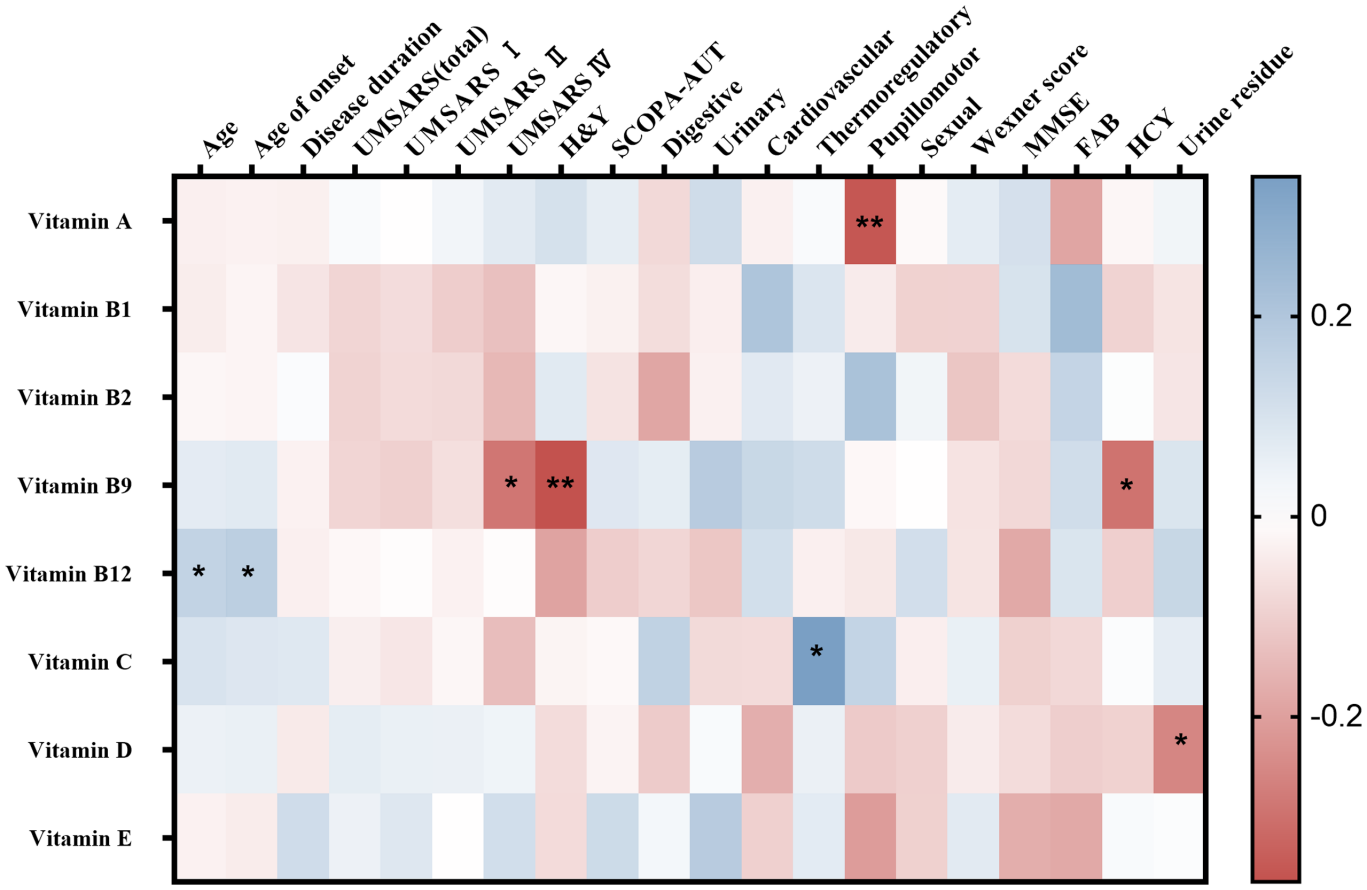
Heatmap for correlation analysis between different vitamins and clinical characteristics of multiple system atrophy (MSA). UMSARS, the Unified MSA Rating Scale; H&Y, Hoehn and Yahr Parkinson’s disease staging scheme; SCOPA-AUT, the Scales for Outcomes in PD-Autonomic questionnaire; MMSE, Mini-Mental State Examination score; FAB, Frontal Assessment Battery score. *p < 0.05; **p < 0.01.

Spearman’s correlation analysis was performed in 124 MSA patients to evaluate the correlations between the clinical characteristics and serum vitamin levels (Table 3; Supplementary Table 3). A significant correlation was observed between serum vitamin A and SCOPA-AUT pupillomotor subdomain (*r*_s_ = −0.356, *p* = 0.01). Meanwhile, the serum vitamin B9 levels were found to be negatively correlated with UMSARS IV, H&Y stages, and HCY (*r*_s_ = −0.290 [*p* = 0.001], −0.365 [*p* = 0.11], and −0.297 [*p* = 016], respectively). Additionally, our data showed that serum vitamin C levels positively correlated with SCOPA-AUT thermoregulatory subdomain (*r*_s_ = 0.339, *p* = 0.028). Besides, vitamin D negatively correlated with urine residue. Vitamin B12 positively correlated with age and age of onset. However, the association between vitamin B12 and age of onset showed no significance after adjusting for age. In addition to such findings, no significant correlations were found between vitamins and remaining clinical characteristics (Figure 3).

**Table 3:** Correlations between vitamin A, B9, and C with clinical characteristics of multiple system atrophy (MSA).

Variable	Vitamin A		Vitamin B9		Vitamin C	
	*r*_s_	*p*	*r*_s_	*p*	*r*_s_	*p*
Age	−0.035	0.583	0.053	0.413	0.077	0.229
Age of onset	−0.030	0.637	0.058	0.365	0.068	0.289
Disease duration	−0.033	0.607	−0.030	0.637	0.063	0.329
UMSARS (total)	0.011	0.911	−0.089	0.325	−0.037	0.680
UMSARS I	−0.004	0.964	−0.101	0.261	−0.053	0.562
UMSARS II	0.024	0.795	−0.069	0.446	−0.020	0.823
UMSARS IV	0.057	0.536	−0.290**	0.001	−0.140	0.125
H&Y	0.084	0.570	−0.365*	0.011	−0.025	0.867
SCOPA-AUT	0.048	0.677	0.064	0.582	−0.015	0.897
Digestive	−0.082	0.437	0.050	0.639	0.132	0.209
Urinary	0.099	0.377	0.154	0.171	−0.080	0.480
Cardiovascular	−0.032	0.817	0.113	0.412	−0.078	0.573
Thermoregulatory	0.011	0.943	0.100	0.528	0.339*	0.028
Pupillomotor	−0.356**	0.001	−0.018	0.870	0.124	0.254
Sexual	−0.014	0.904	−0.003	0.980	−0.036	0.762
Wexner score	0.052	0.614	−0.060	0.561	0.042	0.682
MMSE	0.085	0.397	−0.084	0.402	−0.099	0.323
FAB	−0.190	0.083	0.097	0.379	−0.084	0.447
HCY	−0.019	0.879	−0.297*	0.016	0.007	0.953
Urine residue	0.026	0.804	0.074	0.480	0.051	0.626

*r*_s_, Spearman’s rank correlation coefficient; UMSARS, Unified Multiple system atrophy Rating Scale; PDSS, PD Sleep Scale; H&Y, the modified Hoehn and Yahr staging scale; SCOPA-AUT: the Scales for Outcomes in PD-Autonomic; MMSE, mini-mental state examination; FAB, The Frontal Assessment Battery score; HCY, homocysteine.

* *p* < 0.05;

** *p* < 0.01.

### 3.4. The ROC analysis of vitamins in the diagnosis of MSA

**Figure 4:**
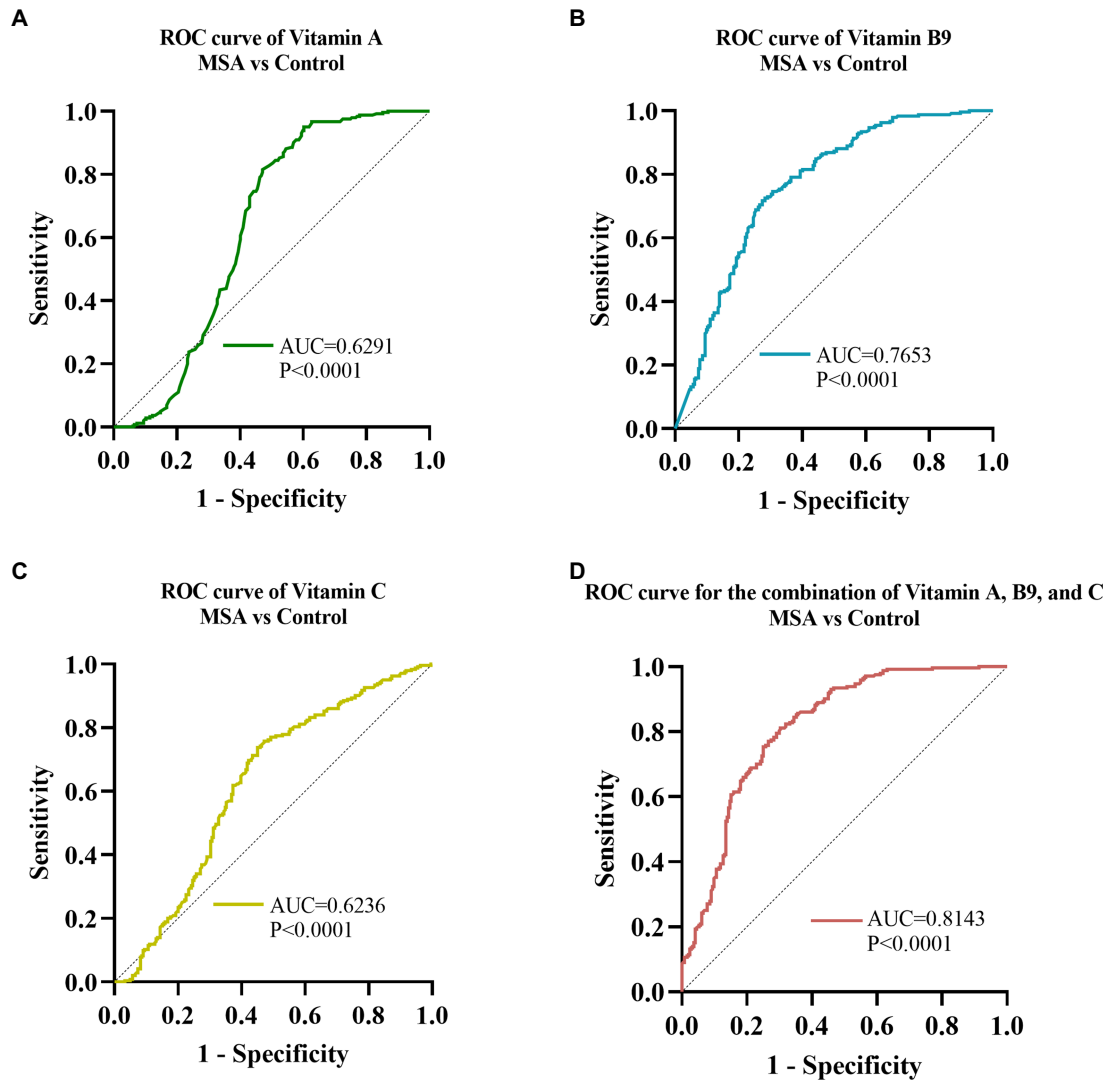
ROC curves to evaluate the utility of serum levels of vitamin for the discrimination of MSA patients from healthy controls. The AUC of ROC curves for **(A)** vitamin A, **(B)** vitamin B9, and **(C)** vitamin C were 0.6291 (95% CI: 0.5769 to 0.6813, *p* < 0.0001), 0.7653 (95% CI: 0.7229 to 0.8077, *p* < 0.001), and 0.6236 (95% CI: 0.5732 to 0.6739, *p* < 0.0001), respectively. The AUC of **(D)** combination of vitamin A, B9, and C was 0.8143 (95% CI: 0.7762 to 0.8523, *p* < 0.001).

Receiver operating characteristic curve (ROC) analysis was conducted to assess the ability of serum vitamins to distinguish between MSA patients and healthy controls. The ROC of vitamin A analysis demonstrated that an area under the curve (AUC) value was 0.6291 (95% CI: 0.5769 to 0.6813, *p* < 0.001, Figure 4A), with a sensitivity of 95% and specificity of 40%. The AUC of vitamin B9 was 0.7653 (95% CI: 0.7229 to 0.8077, *p* < 0.001, Figure 4B), with a sensitivity of 73% and specificity of 72%. The AUC of serum vitamin C was 0.6236 (95% CI: 0.5732 to 0.6739, *p* < 0.001, Figure 4C), with a sensitivity of 74% and specificity of 55%. Furthermore, the optimal ROC curve was provided by the combination of vitamin A, B9, and C for discrimination between MSA patients and healthy controls, of which the AUC was 0.8143 (95% CI: 0.7762 to 0.8523, *p* < 0.001, Figure 4D), with a sensitivity of 81% and specificity of 70%.

## 4. Discussion

Our study included the largest-sample MSA cohorts till now and investigated the variation of serum vitamin levels and their correlation with disease severity in MSA patients. We found the decreased vitamin B9 and increased vitamin A and C levels in MSA patients compared with healthy controls, no matter in male or female. Besides, vitamin A, B9, and C were found to be significantly correlated with motor, pupillomotor, and thermoregulatory dysfunction, respectively. Moreover, the ROC curve analysis demonstrated that the combination of vitamin A, B9, and C could help to discriminate MSA patients from healthy subjects. As far as we know, this study was the most comprehensive research to investigate the changes of the serum vitamins levels in MSA patients, further highlighting the role of vitamins homeostasis dysregulation in MSA pathogenesis.

The limited understanding of the etiology hindered the development of diagnostic and treatment for MSA. Given that the main biological characteristic is the presence of intracellular aggregates of α-syn, MSA and PD are often thought to share similar pathogenesis (Koga et al., 2021). We noted that several studies have indicated that inflammatory responses and oxidative stress played a significant role in the neurodegenerative cascade leading to neurodegenerative diseases (Wyss-Coray and Mucke, 2002; Rebec et al., 2006; Sekiyama et al., 2012; Zhu et al., 2016) There was also evidence that many kinds of vitamins had a close relationship with the occurrence and development of AD and PD (Peterson et al., 2013; Lehmann et al., 2017; Christine et al., 2018; Nourhashemi et al., 2018). What attracted our attention was whether those vitamins were also involved in the pathogenesis of MSA, as well as their relationship with the disease severity.

Vitamin A was involved in several important homeostatic processes, such as cell differentiation, antioxidant activity, inflammation, and neuronal plasticity. The role of vitamin A and its derivatives in the pathogenesis of neurodegenerative diseases, as well as the potentials as therapeutic targets, has drawn attention for years (Eichele, 1997; Clark et al., 2020; Marie et al., 2021). Our study demonstrated a significantly increased vitamin A levels in MSA patients compared with healthy controls, which might due to the compensatory reactions for the dysregulated processes above in MSA. Vitamin C was another elevated vitamin in MSA, contrast to the alteration of serum vitamin C levels in previous studies in PD (Ide et al., 2015; Barmaki et al., 2021). However, it was also reported that serum vitamin C level was found increased in AD patients compared with healthy people (Liu et al., 2022). Oxidative stress was increasingly supposed to be a critical epigenetic factor for neurodegenerative disease and contributed to the aggregation of α-synuclein (Macedo et al., 2015). Vitamin C had a strong capacity for antioxidant and free radical scavenging in serum, central nervous system and other tissues. The elevated serum levels of vitamin A and C further implied that oxidative stress might play an important role in the pathogenesis of the MSA. Further analysis in the sub-group by gender indicated that the alteration of serum levels in the patient with MSA had no relationship with gender.

Interestingly, we observed significantly decreased serum vitamin B9 levels in MSA patients compared with healthy people, while no difference was found in serum vitamin B12 levels between two groups. Consistent results were also obtained in gender-based subgroup analysis. It was known that both vitamin B12 and folate were required in the remethylation of homocysteine (HCY), which was involved in the pathogenesis of neurodegenerative disease *via* mediating inflammation response (Seshadri et al., 2002). Increased serum HCY levels were observed in MSA patients compared with healthy subjects in previous studies (Chen et al., 2015; Guo et al., 2017; Zhong et al., 2022). The decreased vitamin B9 levels might contribute to the neuroinflammation in MSA, which was further validated through the negative correlation between folate and HCY levels. We also observed decreased serum vitamin B9 levels in PD patients compared with healthy controls, which was consistent with the previous meta-analysis result (Shen, 2015; Dong and Wu, 2020). It was proposed that folate deficit might results from the gastrointestinal dysfunction and gut microbial metabolic disorder (Rosario et al., 2021). Similar outcomes were also reported in AD and ALS studies that found decreased serum vitamin B9 level in patients compared with healthy controls (Wang et al., 2020; Liu et al., 2022). The mechanisms of vitamin B9 reduction in MSA was worth exploring. Since the human body could not synthesize folate, exogenously folate supply was necessary. Rosario et al. (2021) found that folate deficit in PD might result from gastrointestinal dysfunction and gut microbial metabolic disorder. The study showed that folate biosynthesis decreased in a state of gastrointestinal dysfunction. Meanwhile, personalized metabolic modeling revealed the microbial contribution to folate deficiency (Rosario et al., 2021). In MSA, the gastrointestinal function was also an important symptom (Sakakibara, 2021). Besides, increasing studies found the presence of gut microbial imbalance in MSA patients, which might serve important roles in MSA pathogenesis. (Tan et al., 2018; Wan et al., 2019). Thus, the gastrointestinal dysfunction and gut microbial dysbiosis might account for the vitamin B9 deficiency in MSA. Overall, the role of vitamin B9 in the pathogenesis of MSA and the potential to treat consequent metabolic and clinical complications warrant more thoroughly investigation.

As for the correlation with clinical scales, vitamin B9 showed negative correlations with UPDRS IV and H&Y scores, indicating that vitamin B9 might influence motor symptoms in MSA patients. Li et al. (2021) demonstrated that serum vitamin B9 and B12 levels were critical factors influencing the motor performance status in PD, where increased serum vitamin B9 level negatively correlated with moderate motor impairment. In addition, our data indicated that serum vitamin A levels were negatively correlated with pupillomotor dysfunction of SCOPA-AUT while vitamin C levels were positively correlated with thermoregulatory dysfunction of SCOPA-AUT scores. Our results suggested that vitamin B9 might have a more prominent influence on motor function of MSA while vitamin A and C were mainly associated with autonomic dysfunction. These results revealed the correlations of vitamins with disease severity of MSA and further implied the critical roles of vitamins in the pathogenic mechanisms of MSA.

ROC curve analysis was performed to assess the ability of serum vitamin A, B9, and C to distinguish MSA patients from HCs. vitamin B9 showed the most reliable diagnostic ability, followed by vitamin A and C. Moreover, our data demonstrated that the combination of vitamin A, B9, and C could provide more reliable discrimination between MSA and healthy controls than any vitamin alone, which might facilitate clinical practice. Notably, the results of the ROC curve analysis did not endorse accurate identification due to the overlap among MSA, PD, and other related diseases, further validation in the larger cohorts were necessary.

The variation between MSA patients and healthy controls provided compromised evidence regarding the involvement of vitamins in the occurrence and development of MSA. Several studies indirectly suggested that vitamins may be useful in the treatment of MSA. *In vitro* experiments indicated that vitamin A could potently destabilize preformed alpha-synuclein fibrils, which suggested that vitamin A might be useful in the treatment and prevention of MSA (Ono and Yamada, 2007). McCarter et al. (2020) found that low vitamin B12 levels were associated with shorter survival in MSA, implying the potential role as a modifiable survival factor of vitamin B12 for MSA. In addition, a systematic review found supplementation of folate had the potential to reduce the high homocysteine concentrations and other clinical complications in Parkinson’s disease (Boelens Keun et al., 2021). Noticeably, few study provided reliable clinical evidence and demonstrated clear effectiveness for vitamins in MSA, and strict randomized clinical trials were necessary in the future to validate the benefits of vitamin supplementation in MSA patients.

Some limitations of the study warrant consideration. Firstly, our study was cross-sectional research and the longitudinal dynamics of serum vitamins levels, as well as the effects on the disease progression, were needed to be further explored in the longitudinal study. Then, the difference of serum vitamins levels between MSA and other easily-confused diseases, such as PSP (Progressive Supranuclear Palsy), and SAOA (Sporadic Adult-Onset Ataxia). remained to be further investigated. Thus, the future longitudinal cohort study including more diseases mimicking MSA are needed to uncover the dysregulation of vitamins homeostasis in MSA pathogenesis thoroughly. Moreover, we did not collect detailed dietary and economical information of the participants in this retrospective study. However, all subjects were from south-central China, who might have similar dietary patterns. Besides, we excluded those who were overweight or malnourished, as well as taking vitamin supplements. In the following studies, we will collect the detailed dietary and economic data through related questionnaires to further validated our conclusions.

In conclusion, based on the case-control study in the largest MSA cohort so far, we revealed the significant changes in the blood vitamin spectrums of MSA patients, suggesting the pivotal role of dysregulation of vitamins homeostasis in the pathogenesis of MSA. The present study showed that several vitamins level were altered and associated with the severity of disease in MSA patients, demonstrating a potential value for disease diagnosis and surveillance. Our data also provided supports for clinical guidance on vitamin supplements of MSA patients. Further validations and explorations in the longitudinal cohorts consisting of more disease mimicking MSA were necessary.

## Data availability statement

The original contributions presented in the study are included in the article/Supplementary material; further inquiries can be directed to the corresponding author.

## Ethics statement

The studies involving human participants were reviewed and approved by the Ethics Committee of the Xiangya Hospital of Central South University. The patients/participants provided their written informed consent to participate in this study.

## Author contributions

DC and LW designed the study. DC analyzed data and wrote original draft. DC, XY, ML, and XZ contributed to the data acquisition of vitamin levels. ZT, XY, DC, YF, and SZ collected clinical data. LW, ZC, RQ, BT, and HJ supervised the process and edited the manuscript. All authors contributed to the article and approved the submitted version.

## Funding

This study was funded by the National Key R&D Program of China (No. 2021YFA0805200 to HJ), the National Natural Science Foundation of China (No. 81974176 and No. 82171254 to HJ; No. 81901169 to ZC; No. 81901305 to CW; No. 82201411 to L He), the Innovation Research Group Project of Natural Science Foundation of Hunan Province (No. 2020JJ1008 to HJ), the Science and Technology Innovation Group of Hunan Province (No. 2020RC4043 to HJ), the Scientific Research Foundation of Health Commission of Hunan Province (No. B2019183 to HJ), the Key Research and Development Program of Hunan Province (Nos. 2020SK2064 and 2018SK2092 to HJ), the Innovative Research and Development Program of Development and Reform Commission of Hunan Province to HJ, the Natural Science Foundation of Hunan Province (Nos. 2022JJ20094 and 2021JJ40974 to ZC; No. 2020JJ5925 to CW; No. 2022JJ40783 to L He), the Central South University Research Programme of Advanced Interdisciplinary Study (No. 2023QYJC010 to HJ), the Project Program of National Clinical Research Center for Geriatric Disorders (Xiangya Hospital, Nos. 2020LNJJ12 and XYYYJSTG-05 to HJ), the Science and Technology Innovation Program of Hunan Province (2022RC1027 to ZC).

## Conflict of interest

The authors declare that the research was conducted in the absence of any commercial or financial relationships that could be construed as a potential conflict of interest.

## Publisher’s note

All claims expressed in this article are solely those of the authors and do not necessarily represent those of their affiliated organizations, or those of the publisher, the editors and the reviewers. Any product that may be evaluated in this article, or claim that may be made by its manufacturer, is not guaranteed or endorsed by the publisher.
