# Supplementary material for: Serum vitamin levels in multiple system atrophy: A case-control study
Source: Front Aging Neurosci. 2023 Jan 5;14:1105019. doi: 10.3389/fnagi.2022.1105019 (PMC9849558; doi:10.3389/fnagi.2022.1105019)
Supplement: Supplementary file 1 [file Table_1.DOCX]

Supplementary Material

Supplementary Table 1: Comparison of serum vitamin level between healthy controls and MSA patients according to genders

|  | MSA, mean(SD) | |  | Control, mean(SD) | |  | P | |
| --- | --- | --- | --- | --- | --- | --- | --- | --- |
|  | male | female |  | male | female |  | P _m_ | P _F_ |
| Age | 56.54 ± 7.97 | 56.60 ± 6.91 |  | 56.23 ± 8.77 | 55.80 ± 7.14 |  | 0.538 | 0.370 |
| vitamin A(umol/L) | 2.57 ± 0.65 | 2.51 ± 0.73 |  | 2.26 ± 0.29 | 2.27 ± 0.21 |  | <0.001 | 0.024 |
| vitamin B1(nmol/L) | 127.19 ± 19.30 | 126.18 ± 1.43 |  | 127.47 ± 14.49 | 127.94 ± 13.53 |  | 0.640 | 0.930 |
| vitamin B2(ug/L) | 7.96 ± 1.27 | 8.06 ± 1.45 |  | 7.93 ± 1.05 | 8.05 ± 1.07 |  | 0.584 | 0.174 |
| vitamin B9 (ug/L) | 9.28 ± 5.17 | 10.40 ± 6.38 |  | 13.84 ± 4.92 | 16.84 ± 5.23 |  | <0.001 | <0.001 |
| vitamin B12(ng/L) | 490.62 ± 327.85 | 545.72 ± 355.70 |  | 489.90 ± 212.19 | 525.67 ± 223.97 |  | 0.055 | 0.317 |
| vitamin C(umol/L) | 31.70 ± 8.54 | 32.75 ± 9.11 |  | 28.55 ± 5.87 | 28.62 ± 7.31 |  | 0.001 | 0.001 |
| vitamin D(nmol/L) | 87.65 ± 26.03 | 87.00 ± 30.99 |  | 87.64 ± 15.41 | 87.68 ± 15.83 |  | 0.254 | 0.209 |
| vitamin E(ug/mL) | 7.05 ± 1.14 | 7.05 ± 1.34 |  | 7.10 ± 0.83 | 6.96 ± 0.81 |  | 0.263 | 0.777 |

Mann–Whitney U-test for the comparison among MSA and normal subjects. Pm, comparison between MSA patients and controls in male; PF, comparison between MSA patients and controls in female

Supplementary Table 2: Comparison of serum vitamin level between MSA-C and MSA-P subgroups.

|  | MSA-C (149) | MSA-P (95) | Crude OR (95%CI) | P_c_ | Adjust OR (95%CI) | P_a_ |
| --- | --- | --- | --- | --- | --- | --- |
| vitamin A(umol/L) | 2.56 ± 0.66 | 2.52 ± 0.74 | 1.078 (0.730, 1.592) | 0.705 | 1.036 (0.694, 1.547) | 0.862 |
| vitamin B1(nmol/L) | 126.00 ± 20.48 | 127.99 ± 19.68 | 0.994 (0.981, 1.008) | 0.411 | 0.991 (0.977, 1.006) | 0.234 |
| vitamin B2(ug/L) | 7.96 ± 1.48 | 7.80 ± 1.10 | 1.118 (0.910, 1.373) | 0.289 | 1.135 (0.915, 1.408) | 0.249 |
| folate(ug/L) | 9.52 ± 5.31 | 10.08 ± 6.30 | 0.983 (0.939, 1.030) | 0.482 | 0.987 (0.941, 1.035) | 0.589 |
| vitamin B12(ng/L) | 510.47 ± 335.79 | 504.07 ± 347.74 | 1.000 (0.999, 1.001) | 0.695 | 1.000 (1.000,1.001) | 0.303 |
| vitamin C(umol/L) | 31.87 ± 8.90 | 32.52 ±8.62 | 0.992 (0.963, 1.023) | 0.619 | 0.996 (0.965, 1.028) | 0.799 |
| vitamin D(nmol/L) | 86.65 ± 28.12 | 88.56 ± 28.16 | 0.997 (0.988, 1.007) | 0.581 | 0.999 (0.988, 1.009) | 0.773 |
| vitamin E(ug/mL) | 7.08 ± 1.22 | 7.00 ± 1.22 | 1.043 (0.833, 1.306) | 0.713 | 1.027 (0.814, 1.295) | 0.824 |

binary logistic regression analysis for the comparison between two subgroups of MSA patients. OR: odds ratio, Pc: P-value for crude OR; Pa: P-value for adjusted OR.

Supplementary Table 3: Correlation analysis between different vitamins and clinical characteristics of multiple system atrophy (MSA).

| **Variable** | vitamin B1 | |  | vitamin B2 | |  | vitamin B12 | |  | vitamin D | |  | vitamin E | |
| --- | --- | --- | --- | --- | --- | --- | --- | --- | --- | --- | --- | --- | --- | --- |
|  | *r_s_* | *P* |  | *r_s_* | *P* |  | *r_s_* | *P* |  | *r_s_* | *P* |  | *r_s_* | *P* |
| Age | -0.040 | 0.532 |  | -0.019 | 0.766 |  | 0.128* | 0.046 |  | 0.037 | 0.564 |  | -0.030 | 0.645 |
| Age of onset | -0.024 | 0.708 |  | -0.024 | 0.709 |  | 0.142* | 0.026 |  | 0.041 | 0.526 |  | -0.042 | 0.510 |
| Disease duration | -0.057 | 0.375 |  | 0.008 | 0.906 |  | -0.035 | 0.586 |  | -0.046 | 0.470 |  | 0.098 | 0.129 |
| UMSARS (total) | -0.090 | 0.321 |  | -0.093 | 0.307 |  | -0.017 | 0.848 |  | 0.050 | 0.583 |  | 0.036 | 0.694 |
| UMSARS Ⅰ | -0.074 | 0.416 |  | -0.077 | 0.396 |  | -0.007 | 0.935 |  | 0.041 | 0.654 |  | 0.066 | 0.463 |
| UMSARS Ⅱ | -0.107 | 0.238 |  | -0.081 | 0.371 |  | -0.030 | 0.739 |  | 0.039 | 0.671 |  | -0.004 | 0.961 |
| UMSARS Ⅳ | -0.134 | 0.143 |  | -0.151 | 0.099 |  | -0.007 | 0.944 |  | 0.029 | 0.755 |  | 0.093 | 0.312 |
| H&Y | -0.019 | 0.896 |  | 0.058 | 0.695 |  | -0.195 | 0.185 |  | -0.075 | 0.613 |  | -0.078 | 0.598 |
| SCOPA-AUT | -0.031 | 0.787 |  | -0.061 | 0.600 |  | -0.106 | 0.358 |  | -0.026 | 0.822 |  | 0.103 | 0.374 |
| Digestive | -0.073 | 0.476 |  | -0.189 | 0.071 |  | -0.088 | 0.406 |  | -0.110 | 0.296 |  | 0.021 | 0.845 |
| Urinary | -0.036 | 0.752 |  | -0.032 | 0.779 |  | -0.120 | 0.288 |  | 0.012 | 0.917 |  | 0.154 | 0.171 |
| Cardiovascular | 0.176 | 0.197 |  | 0.060 | 0.664 |  | 0.090 | 0.511 |  | -0.171 | 0.211 |  | -0.101 | 0.465 |
| Thermoregulatory | 0.071 | 0.653 |  | 0.035 | 0.326 |  | -0.035 | 0.327 |  | 0.039 | 0.671 |  | 0.055 | 0.728 |
| Pupillomotor | -0.043 | 0.694 |  | 0.188 | 0.081 |  | -0.050 | 0.648 |  | -0.112 | 0.300 |  | -0.210 | 0.051 |
| Sexual | -0.097 | 0.417 |  | 0.026 | 0.280 |  | 0.091 | 0.447 |  | -0.100 | 0.401 |  | -0.099 | 0.407 |
| Wexner score | -0.095 | 0.356 |  | -0.122 | 0.235 |  | -0.058 | 0.571 |  | -0.043 | 0.674 |  | 0.057 | 0.578 |
| MMSE | 0.079 | 0.432 |  | -0.077 | 0.444 |  | -0.183 | 0.066 |  | -0.075 | 0.454 |  | -0.172 | 0.085 |
| FAB | 0.211 | 0.054 |  | 0.123 | 0.267 |  | 0.074 | 0.505 |  | -0.104 | 0.345 |  | -0.183 | 0.096 |
| HCY | -0.094 | 0.644 |  | 0.005 | 0.970 |  | -0.103 | 0.413 |  | -0.097 | 0.442 |  | 0.012 | 0.922 |
| Urine residue | -0.058 | 0.368 |  | -0.055 | 0.602 |  | 0.116 | 0.269 |  | -0.256* | 0.013 |  | 0.007 | 0.943 |

rs, Spearman’s rank correlation coefficient; UMSARS, Unified Multiple system atrophy Rating Scale; PDSS, PD Sleep Scale; H&Y, the modified Hoehn and Yahr staging scale; SCOPA-AUT: the Scales for Outcomes in PD-Autonomic; MMSE, mini-mental state examination; FAB: The Frontal Assessment Battery score; HCY: homocysteine

*p<0.05
